# Correlation of the Osteoarthritis Susceptibility Variants That Map to Chromosome 20q13 With an Expression Quantitative Trait Locus Operating on *NCOA3* and With Functional Variation at the Polymorphism rs116855380

**DOI:** 10.1002/art.39278

**Published:** 2015-10-28

**Authors:** Fiona Gee, Michael D. Rushton, John Loughlin, Louise N. Reynard

**Affiliations:** ^1^ Newcastle University Newcastle upon TyneUK

## Abstract

**Objective:**

To functionally characterize the osteoarthritis (OA) susceptibility variants that map to a region of high linkage disequilibrium (LD) on chromosome 20q13 marked by the single‐nucleotide polymorphism (SNP) rs6094710 and encompassing *NCOA3* and *SULF2*.

**Methods:**

Nucleic acids were extracted from the cartilage of OA patients. Overall and allelic expression of *NCOA3* and *SULF2* were measured by quantitative reverse transcription–polymerase chain reaction and pyrosequencing, respectively. The functional effect of SNPs within the 20q13 locus was assessed in vitro using luciferase reporter constructs and electrophoretic mobility shift assays (EMSAs). The in vivo effect of nuclear receptor coactivator 3 (NCOA3) protein depletion on primary human OA articular cartilage chondrocytes was assessed using RNA interference.

**Results:**

Expression of *NCOA3* correlated with the genotype at rs6094710 (*P* = 0.006), and the gene demonstrated allelic expression imbalance (AEI) in individuals heterozygous for the SNP (mean AEI 1.21; *P* < 0.0001). In both instances, expression of the OA‐associated allele was reduced. In addition, there was reduced enhancer activity of the OA‐associated allele of rs116855380, a SNP in perfect LD with rs6094710 in luciferase assays (*P* < 0.001). EMSAs demonstrated a protein complex binding with reduced affinity to this allele. Depletion of NCOA3 led to significant changes (all *P* < 0.05) in the expression of genes involved in cartilage homeostasis.

**Conclusion:**

*NCOA3* is subject to a *cis*‐acting expression quantitative trait locus in articular cartilage, which correlates with the OA association signal and with the OA‐associated allele of the functional SNP rs116855380, a SNP that is located only 10.3 kb upstream of *NCOA3*. These findings elucidate the effect of the association of the 20q13 region on OA cartilage and provide compelling evidence of a potentially causal candidate SNP.

Osteoarthritis (OA) is a common, multifactorial disease characterized by progressive focal loss of articular cartilage, which can be accompanied by changes in the function of other tissues in the joint 1. Genetics is a major risk factor for OA and acts via a large number of susceptibility alleles of small individual effect sizes 2.

Several recent genome‐wide association studies (GWAS) have identified susceptibility loci for OA 3, 4, 5, including a recent meta‐analysis of >78,000 European subjects in whom a genome‐wide significant signal associated with hip OA was identified 6. This signal, at chromosome 20q13, was marked by the single‐nucleotide polymorphism (SNP) rs6094710 (G/A) (*P* = 7.9 × 10^− 9^; odds ratio 1.28). This SNP is moderately rare, with a minor allele frequency (MAF) of 0.04 (4%) in Europeans. It is located upstream of *NCOA3* and *SULF2*, genes that encode nuclear receptor coactivator 3 and extracellular heparan sulfate 6‐O‐endosulfatase 2, respectively. Expression of *NCOA3* is reduced in OA cartilage compared to preserved cartilage from the same joint 6, whereas *SULF2* expression is increased in OA cartilage compared to normal articular cartilage 7.

The SNP rs6094710 is in perfect linkage disequilibrium (LD) (r^2^ = 1, D′ = 1) with 11 other SNPs within the 20q13 region. There are no other SNPs with an r^2^ value of >0.6 with these 12 SNPs. This strongly implies that the association signal is mediated by 1 of the 12 SNPs, all of which encompass a region of 194 kb that includes all of *NCOA3* and approximately two‐thirds of *SULF2*. One of the 12 SNPs, rs6094752, is a missense SNP leading to an amino acid change (Arg>Cys) at position 218 in the NCOA3 protein 6. However, this substitution is predicted to be benign, according to a study in which prediction tools were used 8.

The vast majority of risk alleles for common diseases modulate susceptibility by acting as expression quantitative trait loci (eQTLs), which influence the expression or stability of a transcript 9, 10, 11. In OA, an excellent example is rs143383, which is located in the 5 ′‐untranslated region of *GDF5*; the T allele of this SNP correlates with reduced *GDF5* expression in the joint tissue of OA patients 12. We hypothesized, therefore, that the association with OA susceptibility marked by rs6094710 is an eQTL that acts on *NCOA3* and/or *SULF2*, and the functionality of this eQTL can be directly linked to either rs6094710 or 1 of the 11 SNPs that are in perfect LD with it. We tested this hypothesis by examining both overall gene expression and allelic expression of *NCOA3* and *SULF2* in cartilage tissue from OA patients, and then used a number of experimental methods to further pursue the identified functionality.

## PATIENTS AND METHODS

### Patients

The Newcastle and North Tyneside research ethics committee (REC) granted ethics approval (REC reference no. 09/H0906/72) to obtain cartilage tissue from patients with primary OA undergoing elective hip or knee replacement. Informed consent was obtained from each donor. Full‐thickness, macroscopically normal articular cartilage away from the OA lesion was collected. Further information regarding the 65 patients is provided in Supplementary Table 1 (available on the *Arthritis & Rheumatology* web site at http://onlinelibrary.wiley.com/doi/10.1002/art.39278/abstract). Nucleic acids were extracted and complementary DNA (cDNA) was synthesized as previously described 13, 14.

### Evaluation of gene expression

Gene expression was assessed by quantitative real‐time reverse transcription–polymerase chain reaction (RT‐PCR) using an ABI Prism 7900HT Sequence Detection System (Applied Biosystems). *NCOA3* reactions were performed using a TaqMan Gene Expression Assay (Hs01105251_m1; Applied Biosystems). All other reactions were performed using PrimeTime quantitative PCR assays (Integrated DNA Technologies). A list of the primer and probe sequences used for RT‐PCR is available in Supplementary Table 2 (available on the *Arthritis & Rheumatology* web site at http://onlinelibrary.wiley.com/doi/10.1002/art.39278/abstract). For each cDNA sample, 3 pipetting replicates were performed to determine the expression levels of each gene. These expression levels, relative to those of the housekeeping genes *18S*, *GAPDH*, and *HPRT1*, were calculated using the formula $2^{-\Delta C_{t}}$. Outliers were removed from the data using Grubbs’ test 15. *P* values were calculated using a Mann‐Whitney 2‐tailed exact test.

### Transcript SNP selection and allelic expression analysis

The SNP rs6094752 was used for *NCOA3*, since this transcript SNP is in perfect LD with rs6094710. It is located in exon 7 of the gene. The SNP rs3810526 was used for *SULF2*, since this transcript SNP has the highest pairwise LD with rs6094710 (r^2^ = 0.006, D′ = 0.40). The SNP rs3810526 is a G/A amino acid transition, has an MAF of 0.34 in Europeans, and is located in exon 3 of *SULF2*.

Allelic expression imbalance (AEI) was determined as previously described 16. Standard PCR, using a G‐Storm GS4 Q4 Quad Block Thermal Cycler (Somerton Biotechnology Centre), was used to amplify the rs6094752 and rs3810526 regions with biotinylated primers, creating biotinylated PCR products. Samples were then analyzed on a PyroMark Q24 MDx platform (Qiagen) using a PyroMark Gold Q96 Reagents kit, in accordance with the manufacturer's instructions. The primers used for each SNP, including the sequencing primers, were obtained from Sigma‐Aldrich (a list is provided in Supplementary Table 2, available on the *Arthritis & Rheumatology* web site at http://onlinelibrary.wiley.com/doi/10.1002/art.39278/abstract) and were designed to reside within single exons. Sequences were generated automatically, and an output of allelic ratio was produced using PSQ 96 SQA software (Qiagen).

For each patient, samples were analyzed in triplicate, cartilage cDNA and cartilage DNA were analyzed concurrently, and allelic expression of cDNA was normalized to that of its corresponding DNA. Accurate discrimination of SNP alleles was verified using artificially created allelic expression ratios derived from DNAs of known genotype. *P* values for each SNP were calculated by comparing DNA allelic expression ratios to cDNA allelic expression ratios. Comparisons were made using a Mann‐Whitney 2‐tailed exact test. In addition, pyrosequencing was used to genotype rs6094710.

### Construction of luciferase reporter plasmids, cell cultures, and luciferase assays

Two online databases were searched to identify SNPs within the 20q13 region that are in LD with rs6094710: the Broad SNAP database, which encompasses data from the 1000 Genomes project (http://www.broadinstitute.org/mpg/snap/), and the HapMap database (http://hapmap.ncbi.nlm.nih.gov). To generate the constructs for use in the luciferase reporter gene assay, primers containing either an *Mlu* I or an *Xho* I restriction enzyme site toward their 5 ′ ends were designed to amplify DNA regions encompassing each of the 12 SNPs. Primer sequences are listed in Supplementary Table 3 (available on the *Arthritis & Rheumatology* web site at http://onlinelibrary.wiley.com/doi/10.1002/art.39278/abstract). Template DNA consisted of a mix of DNA from 4 patients heterozygous for rs6094710.

PCR products were purified using a QIAquick PCR purification kit (Qiagen) and cloned into the pGL3‐promoter luciferase reporter plasmid (Promega) using the *Mlu* I and *Xho* I restriction sites. Positive clones were sequenced to ensure the correct sequence of the constructs. The SW1353 human chondrosarcoma cell line and the SW872 human liposarcoma cell line (both from ATCC) were transfected for the luciferase assays. Twenty‐four hours before transfection, SW1353 cells were seeded at a density of 6,000 per well, while SW872 cells were seeded at a density of 10,000 per well, in a 96‐well plate. Cells were transfected with 50 ng of pGL3 construct DNA and 30 ng *Renilla* luciferase reporter vector pRL‐TK (Promega), using FuGene HD transfection reagent (Promega). After 24 hours, the cells were lysed and luciferase activity was determined using a Dual‐Luciferase Reporter Assay System (Promega). Luminescence was measured using a GloMax‐Multi Detection System (Promega). Firefly luciferase activity was normalized to the activity of *Renilla* luciferase. Six technical and 6 biologic repeats were performed per cell line per construct. *P* values were calculated using a Mann‐Whitney 2‐tailed exact test.

### Electrophoretic mobility shift assays (EMSAs)

The JASPAR, PROMO, and TFSearch online databases were used to predict protein binding to the A and G alleles of rs116855380 17, 18, 19. Nuclear protein was extracted from SW1353 and SW872 cells as previously described 20. Forward and reverse single‐stranded DY682‐labeled oligonucleotides (Eurofins MWG Operon) for both alleles, spanning 15 bp to each side of rs116855380, were annealed to generate double‐stranded probes, and EMSAs were performed as previously described 20.

All EMSA experiments were performed twice. Briefly, binding reactions were performed for 20 minutes at room temperature using an Odyssey EMSA buffer kit (LiCor Biosciences). Each reaction contained 5 μg nuclear extract, 200 fmoles annealed oligonucleotide, 1 × Binding Buffer, 2.5 m*M* dithiothreitol, 0.5 μg poly(dI‐dC), and 10 m*M* EDTA. Samples were separated on a 5% (weight/volume) native polyacrylamide gel in 0.5 × Tris–borate–EDTA for 4 hours at 100 volts, followed by visualization with an Odyssey Infrared Imager (LiCor Biosciences). For competition assays, unlabeled oligonucleotides identical to the probes, or containing a transcription factor consensus sequence, were added to the binding reaction in excess. For supershift assays, 2 μg or 6 μg of antibody was added to the binding reaction. All probe and competitor sequences and antibodies used in the EMSAs are listed in Supplementary Tables 4 and 5 (available on the *Arthritis & Rheumatology* web site at http://onlinelibrary.wiley.com/doi/10.1002/art.39278/abstract).

### Immunohistochemistry

Macroscopically normal cartilage away from the OA lesion was fixed overnight in 10% formalin (Sigma‐Aldrich) and wax embedded. Sections were cut and stained using anti‐NCOA3 antibody (ab133611; Abcam). Briefly, sections were incubated in boiling citrate, pH 6.0, for 6 minutes, washed in Tris buffered saline for 5 minutes, and stained using an ImmPRESS Anti‐Rabbit Ig Reagent Kit (Vector Laboratories), in accordance with the manufacturer's instructions. Primary antibody was applied for 30 minutes at a 1:150 dilution, diaminobenzidine was used as the peroxidase substrate, and hematoxylin was used to counterstain.

### RNA interference

Primary human articular chondrocytes (HACs) were isolated by enzymatic digestion of OA cartilage and cultured as described previously 21. HACs were seeded 24 hours before transfection with 100 n*M* Dharmacon ON‐TARGETplus SMARTpool small interfering RNA (siRNA) targeted against *NCOA3* (L‐003759‐00) or an ON‐TARGETplus nontargeting siRNA control pool (D‐001810‐10‐20), using DharmaFECT 1 transfection reagent (Dharmacon). After 48 hours, gene expression was assessed by RT‐PCR, and protein levels were assessed by immunoblotting. For RT‐PCR, cells were seeded at a density of 10,000 cells per well in a 96‐well plate, with 6 technical repeats per treatment. Forty‐eight hours after transfection, the 96‐well plate was used to synthesize cDNA using a Cells‐to‐cDNA II kit (Ambion), and gene expression was assessed by RT‐PCR.

For immunoblot analysis, cells were seeded at a density of 350,000 cells per well in a 6‐well plate. Two wells were seeded per treatment and their contents combined before protein extraction. Total protein was extracted from the 6‐well plate using a spin column extraction kit (Nucleospin RNA/Protein; Macherey‐Nagel, supplied by Fisher), in accordance with the manufacturer's instructions. Three biologic repeats were performed using cells obtained from 3 individual OA patients. *P* values were calculated using a Student's 2‐tailed *t*‐test.

### Immunoblotting

To assess depletion of NCOA3, total protein from HACs was quantified (Bradford reagent; Expedeon) and 10 μg was resolved on 12% (w/v) sodium dodecyl sulfate–polyacrylamide gels. Protein was transferred to Immobilon‐P PVDF membranes (Merck Millipore). Anti‐NCOA3 antibody (ab133611; Abcam) was used at a 1:1,000 dilution to assess protein levels. A β‐actin antibody (A5316; Sigma) was used at a 1:10,000 dilution as a loading control. Depletion of NCOA3 was quantified using ImageJ software 22, and NCOA3 levels were normalized to the levels of β‐actin for each treatment condition.

## RESULTS

### Quantitative expression of *NCOA3* and *SULF2* in OA cartilage stratified by rs6094710 genotype

To determine whether the 20q13 locus harbors a cartilage eQTL that correlates with the OA association signal, we quantified the expression levels of *NCOA3* and *SULF2* in articular cartilage samples from patients with OA and stratified the data by genotype at rs6094710. Although rs6094710 is associated only with hip OA, we performed our analyses in both hip and knee tissue in order to determine whether the functional variant operates in knee as well as hip tissue. Since this SNP has a moderately low MAF (0.04), none of our patients were homozygous for the minor allele of the SNP. Our comparisons therefore involved an analysis of GG homozygotes versus GA heterozygotes.

**Figure 1:**
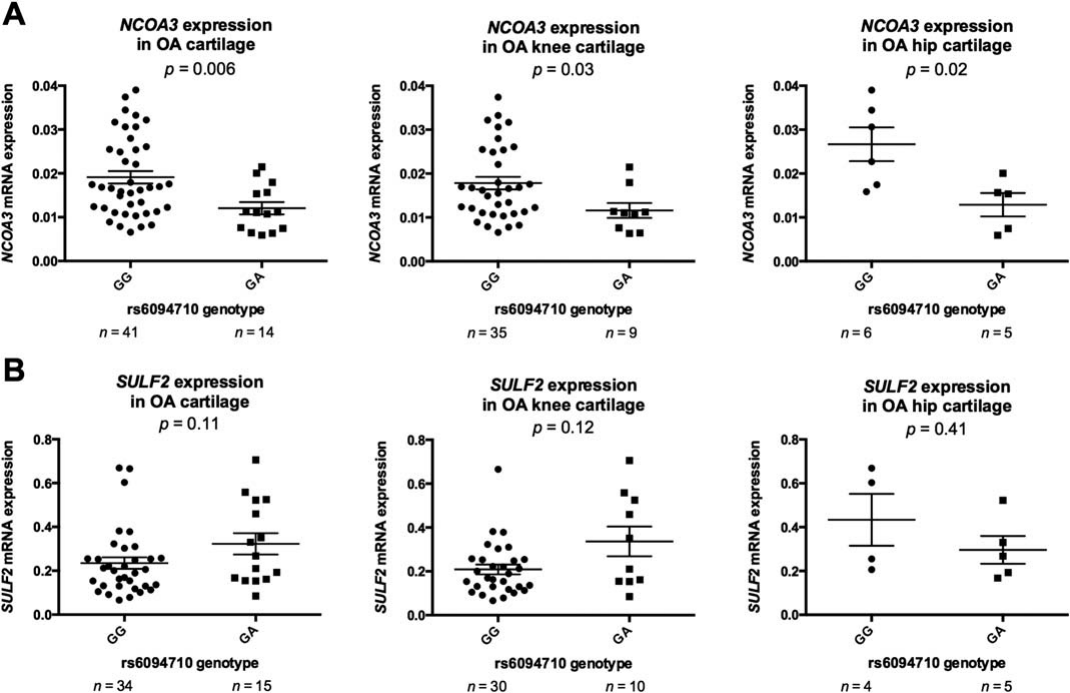
Columnar scatter plots of the quantitative expression of *NCOA3* (**A**) and *SULF2* (**B**) in osteoarthritis (OA) cartilage, stratified by genotype at the OA‐associated single‐nucleotide polymorphism rs6094710. In cartilage samples from all OA patients (left), patients with knee OA (center), and patients with hip OA (right), the expression of each gene was assessed by quantitative real‐time reverse transcription–polymerase chain reaction, with values normalized to the values for the housekeeping genes *18S*, *GAPDH*, and *HPRT1*. Individual symbols represent the average of the 3 replicates for each sample; horizontal lines represent the mean ± SEM. *P* values were calculated by Mann‐Whitney 2‐tailed exact test.

We observed a significant correlation between *NCOA3* expression and genotype (Figure 1A), with presence of the minor A allele of the SNP correlating with reduced gene expression (*P* = 0.006). The A allele of rs6094710 has been found to be more common in OA patients compared to controls 6.

We next separated the OA patients into those with knee OA (Figure 1A) and those with hip OA (Figure 1A). We observed a significant correlation between *NCOA3* expression and genotype both in patients with knee OA and in patients with hip OA (*P* = 0.03 and *P* = 0.02, respectively).

Finally, we stratified *NCOA3* expression by joint site, by sex, and by age at the time of joint replacement. We found no correlations between the level of *NCOA3* expression and any of these 3 parameters (all *P* > 0.05; results in Supplementary Figure 1, available on the *Arthritis & Rheumatology* web site at http://onlinelibrary.wiley.com/doi/10.1002/art.39278/abstract).

There was no correlation between rs6094710 genotype and *SULF2* expression (*P* = 0.11) (Figure 1B). We repeated the analysis after separating patients into those with knee OA (Figure 1B) and those with hip OA (Figure 1B). Again, there was no correlation between rs6094710 genotype and *SULF2* expression in patients with knee OA or those with hip OA (*P* = 0.12 and *P* = 0.41, respectively). When we stratified *SULF2* expression by joint site, by sex, and by age at the time of joint replacement, we found that there was greater gene expression in the hip than in the knee (*P* = 0.04) and there was increased expression of *SULF2* in women compared to men (*P* = 0.04), but there was no correlation with age (*P* = 0.33) (results in Supplementary Figure 1, available on the *Arthritis & Rheumatology* web site at http://onlinelibrary.wiley.com/doi/10.1002/art.39278/abstract).

Overall, our analysis revealed an eQTL operating on *NCOA3* that correlated with the OA association signal. Moreover, we found that the OA risk allele was mediating the reduced expression of the gene.

### AEI analysis of *NCOA3* and *SULF2* in OA cartilage

We further investigated the eQTL by AEI analysis, which measures the messenger RNA (mRNA) output from each allele of a SNP in heterozygous individuals. For *NCOA3*, we used transcript SNP rs6094752, which is in perfect LD (r^2^ = 1, D′ = 1) with the OA‐associated SNP rs6094710. We genotyped 130 patients for whom DNA and RNA samples were available, and identified 8 individuals heterozygous for rs6094710. For *SULF2*, we chose the transcript SNP rs3810526, which has the highest pairwise LD with rs6094710. This LD is nevertheless low (r^2^ = 0.006, D′ = 0.40), and this, along with the low MAF of rs6094710, meant that we were able to identify only 3 compound heterozygous patients among our cohort.

**Figure 2:**
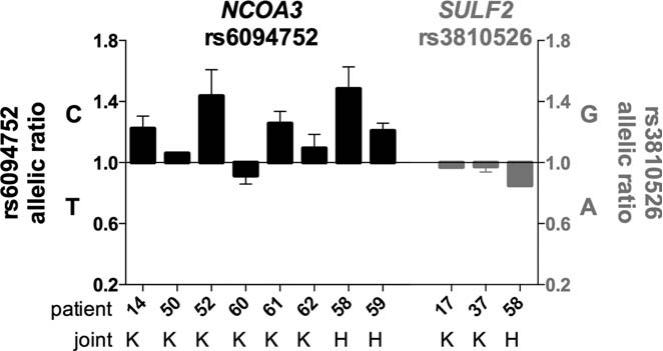
Allelic expression analysis in cartilage samples from osteoarthritis (OA) patients. Allelic expression was assessed using the transcript single‐nucleotide polymorphisms rs6094752 in *NCOA3* (black bars) and rs3810526 in *SULF2* (gray‐shaded bars). Allelic expression of cDNA was normalized to that of its corresponding DNA. Data are presented as a ratio of expression of the major allele over that of the minor allele; thus, a value above 1 means that there is more of the major allele. Samples are grouped according to OA joint site (knee [K] or hip [H]), with the numbers of patients shown. Bars show the mean ± SEM.

Cartilage from 6 patients with knee OA and 2 patients with hip OA was analyzed for AEI at rs6094752, with the T allele (equivalent to the rs6094710 A allele) showing reduced expression in 7 patients (Figure 2). The mean fold difference in allelic expression was 1.21, indicating that the C allele of the SNP correlated with 21% more *NCOA3* expression than the OA‐associated T allele (*P* < 0.0001).

Cartilage from 2 patients with knee OA and 1 patient with hip OA was analyzed for AEI at rs3810526. One of the 3 patients (patient 58) showed increased expression of the A allele (Figure 2). The mean fold difference in allelic expression was 0.93, indicating that the A allele of the SNP produced 7% more *SULF2* than the G allele (*P* = 0.04).

Overall, the AEI data confirmed that the risk allele of the OA association signal correlated with reduced expression of *NCOA3*.

### Identifying rs116855380 as a functional SNP

As already noted, rs6094710 is in perfect LD with 11 SNPs within the 20q13 OA locus (for details regarding the physical location of the SNPs, see Table 1; a visual representation of the locus can be found in Supplementary Figure 2, available on the *Arthritis & Rheumatology* web site at http://onlinelibrary.wiley.com/doi/10.1002/art.39278/abstract). There are no other SNPs within the region showing LD at an r^2^ value of >0.6. To assess whether rs6094710 or any of the other 11 SNPs are functional, we cloned the regions of DNA containing each SNP separately into a luciferase reporter plasmid. We created both allelic forms for each SNP and performed reporter gene expression assays using the SW1353 and SW872 cell lines.

**Table 1:** Luciferase gene reporter assays in SW1353 and SW872 cell lines of the single‐nucleotide polymorphisms (SNPs) in perfect linkage disequilibrium with rs6094710*

SNP, allele (major, minor)	SNP location	Predicted enhancer	SW1353 cells			SW872 cells		
			Luciferase activity	Fold difference (major/minor)	*P*	Luciferase activity	Fold difference (major/minor)	*P*
rs6094710								
G	Intergenic	Yes	3.8	1.1	0.79	5.3	1.0	0.87
A			3.5			5.1		
rs73122077								
C	Intergenic	Yes	1.0	1.0	0.32	1.4	1.1	0.015
T			1.0			1.3		
rs6090683								
G	Intergenic	Yes	1.3	1.1	0.67	1.4	1.2	0.069
C			1.2			1.2		
rs6090684	Intergenic	Yes		1.3	0.013		0.9	0.043
G			0.9			0.9	
A			0.7			1.0	
rs116855380								
A	Intergenic	Yes	1.4	1.2	0.0003	1.6	1.5	<0.0001
G			1.2			1.1		
rs117212926								
G	Intronic (*NCOA3*)	No	0.6	0.9	0.53	1.3	0.9	0.71
A			0.7			1.5		
rs72662711								
T	Intronic (*NCOA3*)	Yes	1.1	1.0	0.99	2.0	1.5	0.0014
C			1.0			1.3		
rs6090704								
G	Intronic (*NCOA3*)	Yes	1.0	0.9	0.61	1.6	1.2	0.027
A			1.1			1.3		
rs6094752								
C	Exonic (*NCOA3*)	No	0.8	1.1	0.70	2.0	1.1	0.33
T			0.8			1.8		
rs72645259								
T	Intronic (*NCOA3*)	No	2.3	0.9	0.34	2.6	0.7	0.0021
C			2.6			4.0		
rs73913405								
C	Intronic (*SULF2*)	Yes	0.7	1.0	0.15	1.3	1.0	0.76
T			0.6			1.3		
rs73913406								
T	Intronic (*SULF2*)	Yes	1.5	1.5	<0.0001	1.2	1.2	0.13
G			1.0			1.0		

Luciferase activity was calculated as the activity of firefly luciferase relative to *Renilla* luciferase, with results normalized to the values in empty vector. *P* values were calculated by Mann‐Whitney 2‐tailed exact test.

In SW1353 cells, we observed significant allelic differences (*P* < 0.05) in luciferase activity for 3 SNPs, while in SW872 cells, we observed significant differences in luciferase activity between the alleles of 6 SNPs (Table 1). Of all the SNPs tested, only 2 of the SNPs, rs116855380 and rs6090684, showed significant differences in both cell lines. However, the allelic differences observed for rs6090684 acted in opposite directions between the 2 cell lines.

**Figure 3:**
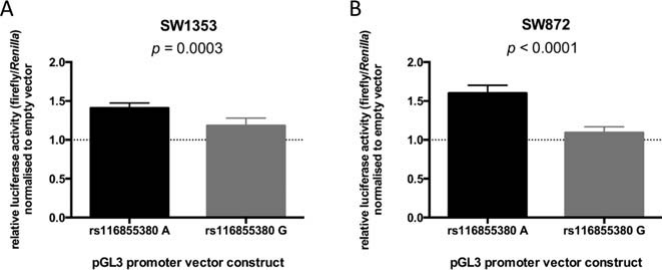
Results of luciferase reporter assays for the major A allele and minor G allele of rs116855380 in SW1353 cells (**A**) and SW872 cells (**B**), assessing firefly luciferase activity relative to *Renilla* luciferase activity, normalized to an empty pGL3‐promoter vector (indicated by the broken line). Bars show the mean ± SEM fold change from 6 biologic repeats, each with 6 technical replicates. *P* values were calculated by Mann‐Whitney 2‐tailed exact test.

The region of DNA containing rs116855380 displayed enhancer activity in both cell lines when the A allele of the SNP was present, and the presence of the G allele was associated with a significant decrease in reporter activity in SW1353 cells (*P* = 0.0003) (Figure 3A) and SW872 cells (*P* < 0.0001) (Figure 3B). The G allele of rs116855380 is in perfect LD with the OA‐associated A allele of rs6094710; thus, these findings from luciferase reporter assays supported the findings from analyses of *NCOA3* expression and AEI.

### EMSA assessment of *trans*‐acting factors binding to rs116855380

**Figure 4:**
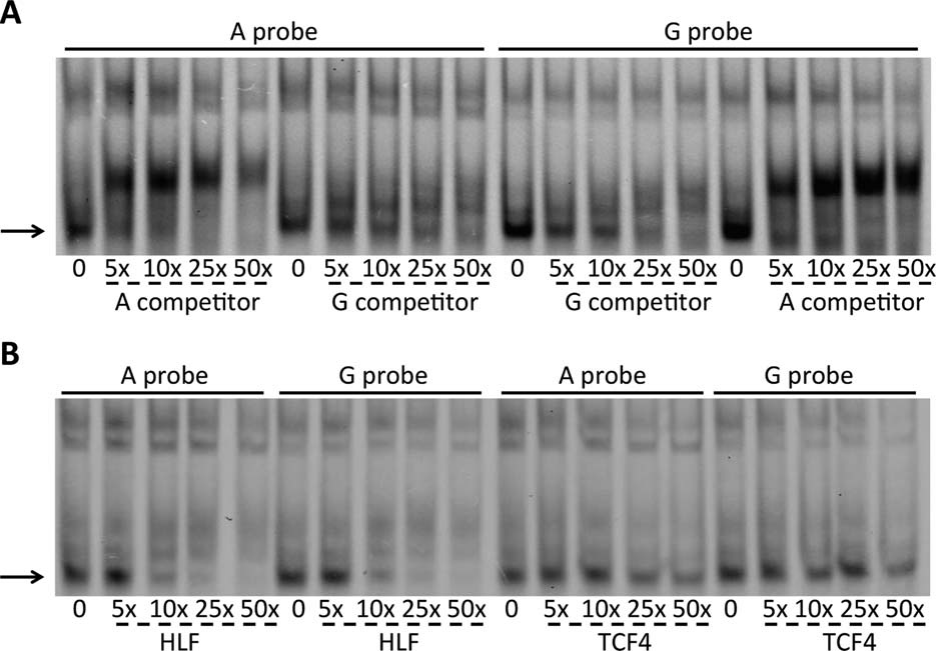
Electrophoretic mobility shift assay (EMSA) analyses in SW1353 cells. **A,** Increasing concentrations of unlabeled A and G allele competitor were added to the EMSA reaction containing cell nuclear extract and the A and G allele probes. **Arrow** indicates the specific complex binding to the probes. **B,** Increasing concentrations of hepatic leukemia factor (HLF) and transcription factor 4 (TCF‐4) unlabeled consensus competitors were added to the EMSA reaction containing the A or G allele probe. **Arrow** indicates the complex that is competed.

An analysis of ENCODE data sets (http://genome.ucsc.edu/ENCODE/) revealed that rs116855380 is predicted to lie within a region of enhancer activity 23, a prediction that was supported by the findings from our luciferase assays. We therefore investigated protein complex binding to rs116855380 using nuclear protein extracted from SW1353 and SW872 cells and using fluorescence‐labeled A and G allele probes representing the 2 alleles of this SNP (results in SW1353 cells shown in Figure 4A; results in SW872 cells shown in Supplementary Figure 3, available on the *Arthritis & Rheumatology* web site at http://onlinelibrary.wiley.com/doi/10.1002/art.39278/abstract).

We observed a similar pattern of protein complex binding to the 2 probes in SW1353 cells. We tested the specificity of the assay by adding unlabeled A and G allele competitors, and found that one complex bound specifically to the rs116855380 probes (Figure 4A). Binding of this complex to both probes was outcompeted with a lower concentration of the A allele competitor than that of the G allele competitor. For example, Figure 4A shows that a lower concentration of the A allele competitor, as compared to the G allele competitor, was required to outcompete the A allele probe. The addition of the A allele competitor at 25× and 50× concentrations resulted in the complete disappearance of the complex (Figure 4A), whereas these same concentrations of the G allele competitor resulted in only a reduction in the intensity of these bands (Figure 4A). The same effect could be observed in SW872 cells and in repeated experiments (results in Supplementary Figure 3, available on the *Arthritis & Rheumatology* web site at http://onlinelibrary.wiley.com/doi/10.1002/art.39278/abstract).

Using the online databases JASPAR, PROMO, and TFSearch, we identified 5 transcription factors (transcription factor 4 [TCF‐4], lymphoid enhancer–binding factor 1 [LEF‐1], hepatic leukemia factor [HLF], serum response factor [SRF], and myocyte enhancer factor 2C [MEF‐2C]) predicted to bind to the region containing rs116855380. We refined the number of potential factors using competitors containing the consensus binding sequence of each factor (the list of competitor sequences is provided in Supplementary Table 4, available on the *Arthritis & Rheumatology* web site at http://onlinelibrary.wiley.com/doi/10.1002/art.39278/abstract). With the addition of an HLF competitor, complex binding to both probes was reduced, and this effect was observed with nuclear extract from both cell lines (results in SW1353 cells shown in Figure 4B; results in SW872 cells shown in Supplementary Figure 3, available on the *Arthritis & Rheumatology* website at http://onlinelibrary.wiley.com/doi/10.1002/art.39278/abstract). In experiments using SW1353 cell nuclear extract, when a TCF‐4 competitor was added, complex binding to both probes was competed (Figure 4B). Little or no competition was observed when the TCF‐4 competitor was used with SW872 cell extract.

Addition of antibodies targeting TCF‐4 or HLF did not result in a supershift of the specific complex (data not shown). Through literature searches, we identified 2 transcription factors closely related to HLF (DNA binding protein and CCAAT/enhancer binding protein) that have very similar consensus binding sites 24. However, addition of antibodies to these proteins also did not result in supershift of the complex (data not shown).

### Regulation of *COL2A1*, *RUNX2*, and *MMP13* transcriptional activity by NCOA3

To assess the presence and distribution of NCOA3 protein, we stained cartilage tissue prepared from nonlesional OA cartilage with an anti‐NCOA3 antibody (details shown in Supplementary Figure 4, available on the *Arthritis & Rheumatology* web site at http://onlinelibrary.wiley.com/doi/10.1002/art.39278/abstract). The protein is present in chondrocytes in both knee and hip cartilage. Cells within the superficial and middle zones stained more strongly than cells in the deep zone. Staining was nuclear, consistent with the role of NCOA3 as a coactivator of various nuclear receptors 25, 26.

**Figure 5:**
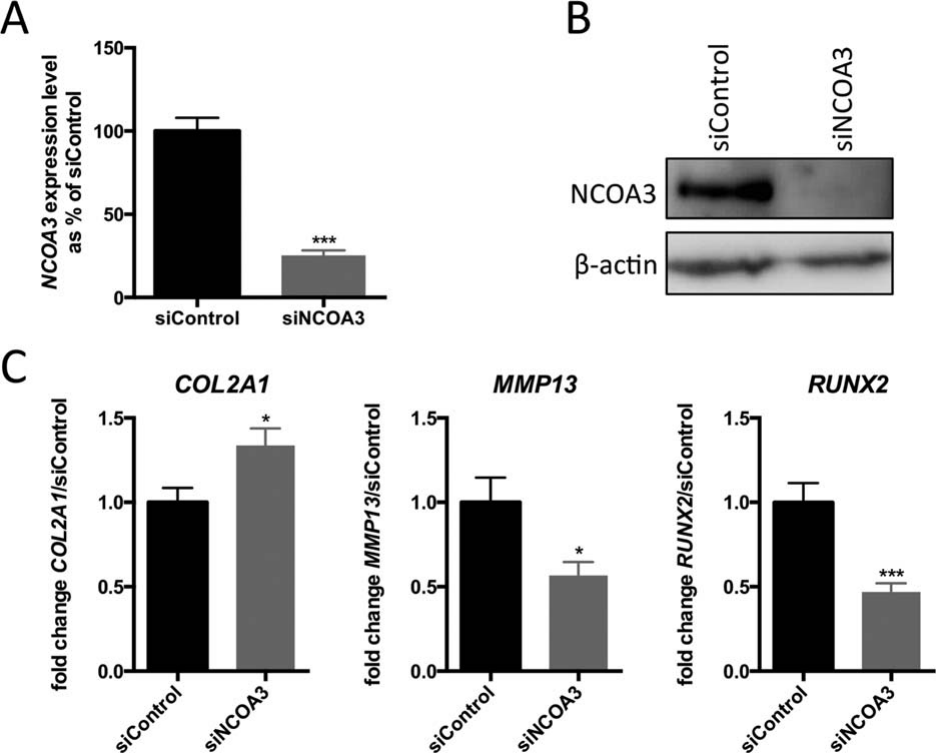
Effects of *NCOA3* depletion on expression of genes involved in cartilage homeostasis. Knockdown of *NCOA3* was performed in primary human articular chondrocytes obtained from 3 patients with osteoarthritis. **A,** Expression levels of *NCOA3* mRNA in cells following *NCOA3* knockdown with small interfering RNA (siRNA) targeting *NCOA3* (siNCOA3). Values are the mean and SEM percentage change in mRNA expression relative to that with the control nontargeting siRNA (siControl) (set at 100%). **B,** Representative immunoblot demonstrating NCOA3 depletion following siNCOA3 treatment; β‐actin was used as a loading control. **C,** Fold change in expression of *COL2A1*, *MMP13*, and *RUNX2* following siNCOA3 treatment. Values are the mean ± SEM fold change relative to that with the siControl (set at 1.0), from 3 biologic repeats, each with 6 technical replicates. *P* values were calculated by Student's 2‐tailed *t*‐test. **∗** = *P* < 0.05; **∗∗∗** = *P* < 0.001 versus siControl.

To investigate the role of NCOA3 in maintaining healthy cartilage, we analyzed the effect of NCOA3 depletion by RNA interference in HACs isolated from nonlesional areas of the OA cartilage. The depletion of *NCOA3* mRNA (75% reduction) was confirmed by RT‐PCR, and the depletion of NCOA3 protein (76% reduction) was confirmed by immunoblotting (Figures 5A and B). We assessed the levels of 5 chondroprotective/anabolic genes (*COL1A1*, *COL2A1*, *ACAN*, *SOX9*, and *TIMP1*) and 4 genes involved in cartilage hypertrophy/catabolism (*RUNX2*, *ADAMTS5*, *MMP1*, and *MMP13*). Expression of *COL2A1* was significantly increased following NCOA3 depletion (*P* = 0.02) (Figure 5C), whereas expression of *MMP13* and *RUNX2* was significantly decreased (*P* = 0.03 and *P* = 0.0002, respectively) (Figure 5C).

## DISCUSSION

In this study, we demonstrated the presence of a cartilage eQTL operating on *NCOA3* that correlates with the OA association signal marked by SNP rs6094710. The OA risk allele, the A allele of the SNP, was associated with reduced expression of the gene. We then uncovered allelic functionality at rs116855380, a SNP in perfect LD with rs6094710; the G allele of rs116855380 segregated on the same molecule as the A allele of rs6094710 in the haplotype that harbors these 2 SNPs. Knockdown of NCOA3 highlighted the important functional role of this protein in chondrocyte biology. As far as we are aware, this is the first study in which an OA association signal derived from a GWAS has been characterized to the point at which the functional effect has been elucidated and a compelling causal candidate SNP identified.

Although rs6094710 has been demonstrated to be associated with OA of the hip, we found that the eQTL operates in both hip and knee cartilage. Many OA risk loci show joint‐specific genetic effects 2, highlighting the complex nature of OA susceptibility and the fact that pathophysiologic causes are not uniform across skeletal sites 27. Presumably, lowered *NCOA3* levels have a markedly detrimental effect in the hip but not in the knee.

The OA risk marked by rs6094710 did not correlate with differential expression of *SULF2*. We did observe a small degree of AEI in 1 of our 3 compound heterozygous patients. We cannot prove a negative, and it may be that there is an eQTL operating on *SULF2*. There is, however, no compelling evidence in our study to indicate that altered expression of this gene correlates with the OA association signal.

The SNP rs116855380, which we discovered as the likely mediator of the *NCOA3* eQTL, is located only 10.3 kb upstream of the gene, in a region that, according to ENCODE, has a transcriptional regulatory function. We investigated *trans*‐acting factors that may bind differentially to the 2 alleles of rs116855380, and which could therefore be involved in mediating the allelic expression imbalance. In EMSA analysis, we showed that the consensus binding sites for TCF‐4 and HLF competed with the rs116855380 probe for binding of a specific complex, which bound with more affinity to the A allele of the SNP. We were, however, unable to observe a supershift. This suggests that another protein with a similar consensus sequence is binding to rs116855380 and promoting transcriptional activation, and that this binding, and therefore activation, is strongest for the A allele of rs116855380.

Other SNPs within the locus showed significant differences in the reporter assays. We therefore cannot discount the possibility that one or more of these SNPs could also have functional effects in the cartilage tissue. However, and unlike rs116855380, the reporter assay effects of these other SNPs were not observed in both cell types examined or in the same direction in both cell types. Our study has therefore enabled us to prioritize rs116855380 as a SNP with consistent in vitro allelic functional effects that match the findings in our OA cartilage tissue.

In our analysis of primary HACs in monolayer, a reduction in NCOA3 protein, which mimics the genetic effect identified, resulted in an increase in *COL2A1* expression and a decrease in *RUNX2* and *MMP13* expression. In this model system, NCOA3 is therefore functional. However, these results are paradoxical when we consider that reduced expression of *NCOA3* is associated with OA risk. Although *COL2A1* expression has been shown to increase in OA cartilage, probably as a compensatory mechanism 28, 29, the expression of *RUNX2* and *MMP13* has also been found to increase 30, 31, with RUNX2 directly up‐regulating *MMP13* 31, 32. Thus, one would expect to see an increase in *RUNX2* and *MMP13* expression when reducing the level of NCOA3 protein in chondrocytes.

Our results are, however, consistent with previous findings that showed that NCOA3 directly regulates transcription of *MMP13* through coactivation of activator protein 1 and polyomavirus enhancer activator 3 33. In addition, as NCOA3 is a coactivator for several nuclear hormone receptors, and *RUNX2* transcription is activated by parathyroid hormone 34, NCOA3 may also act in this capacity to regulate *RUNX2*, and therefore *MMP13*, expression. NCOA3 can also recruit the nuclear factors p300/CREB binding protein–associated factor (P/CAF) and CREB binding protein to create multisubunit coactivator complexes 25, and P/CAF has been shown to increase RUNX2 activity by acetylation 35. To clarify the role of NCOA3 in cartilage biology, more detailed functional analyses are clearly merited, encompassing gene overexpression and knockdown and the use of cells in monolayer and in 3‐dimensional cultures, the latter more accurately reflecting cartilage tissue.

In conclusion, we have identified a SNP and a functional effect that are likely to account for the OA association mapped to chromosome 20q13: rs116855380 and differential allelic expression in *NCOA3* in OA cartilage. Further functional analyses of the SNP, the gene, and its protein are now justified to fully comprehend how this particular genetic risk increases disease susceptibility and how that susceptibility can be mitigated.

## AUTHOR CONTRIBUTIONS

All authors were involved in drafting the article or revising it critically for important intellectual content, and all authors approved the final version to be published. Dr. Reynard had full access to all of the data in the study and takes responsibility for the integrity of the data and the accuracy of the data analysis.

**Study conception and design.** Gee, Rushton, Louglin, Reynard.

**Acquisition of data.** Gee, Rushton.

**Analysis and interpretation of data.** Gee, Rushton, Louglin, Reynard.

## Supporting information

Figure S1. Scatter plots of the quantitative expression of *NCOA3* (left) and *SULF2* (right) in osteoarthritis (OA) cartilage. Data is stratified by (A) joint site, (B) sex and (C) age at surgery. The data points represent the average of the three replicates for each sample. The expression of each gene was assessed by quantitative real‐time reverse transcription PCR and normalized to the housekeeping genes *18S*, *GAPDH* and *HPRT1*. *n* is the number of patients studied. The horizontal lines in the columnar scatter plots represent the mean and the standard error of the mean. The trendlines in the XY scatter plots represent linear regression of the data. *p*‐values for (A) and (B) were calculated using a two‐tailed Mann‐Whitney exact test. *p*‐values for (C) were calculated using linear regression.
Figure S2. Schematic representation of the 20q13 locus. Gene track is a screenshot from http://genome.ucsc.edu, using release hg19. The genes are represented by horizontal lines, with the arrows indicating the direction of gene transcription. The exons are represented by vertical bars, whose width is proportional to the length of the exon. The numbers above the gene tracks indicate the position within chromosome 20. The vertical lines beneath the gene tracks represent the SNPs analyzed in this study. The SNP that identified the signal in the GWAS, rs6094710, is labeled in white text on a black background, and the two transcript SNPs used for AEI, rs6094752 (*NCOA3*) and rs3810526 (*SULF2*), are labeled with **bold** text. Also labeled are the other SNPs in perfect LD with rs6094710.
Figure S3. Electrophoretic mobility shift assay (EMSA) analysis in SW872 (A and B) and SW1353 cells (C and D). (A and C) Increasing concentrations of unlabeled G and A allele competitor were added to the EMSA reaction containing the G and A allele probes and nuclear extract, with the arrow indicating the specific complex binding to the probes. (B and D) The addition of increasing concentrations of HLF and TCF4 unlabeled consensus competitors to the EMSA reaction containing the G or A allele probe. The arrow indicates the complex that is competed.
Figure S4. Expression of NCOA3 in cartilage tissue. (A,B,D,E) Immunohistochemical staining against NCOA3 in macroscopically normal knee (A and D) and hip (B and E) cartilage obtained from osteoarthritis patients. (C and F) Negative control staining with no primary antibody in the same hip sample as shown in B and E. The articular surface is at the top of the images. Top panels (A‐C) were taken at 5x magnification; bottom panels (D‐F) show enlarged views taken at 10x magnification. Cells exhibit nuclear staining, which is consistent with the role of NCOA3 as a co‐activator of various nuclear receptors. Enlarged regions are indicated by the black boxes in A‐C. Scale bars = 200 m.
Table S1. Table of OA patient characteristics, their genotype at rs6094710 and details of their use in quantitative real‐time reverse transcription PCR (RT‐PCR). F, female; M, male; K, knee; H, hip.
Table S2. Primer and probe sequences used for the quantitative real‐time reverse transcription PCR (RT‐PCR) of a panel of genes. The *NCOA3* primers are not listed and were purchased from Applied Biosystems as an off‐the‐shelf TaqMan Gene Expression Assay. Also listed are the primers used for the pyrosequencing analysis of SNPs rs6094710, rs6094752 and rs3810526. FP, forward primer; RP, reverse primer; SP, sequencing primer; Pr, probe
Table S3. The twelve DNA fragments examined by luciferase analysis. Chromosome locations based on UCSC genome browser, release hg19. The sites in the cloning primers for the restriction enzymes MluI (ACGCGT) and XhoI (CTCGAG) are highlighted in bold.
Table S4. Electrophoretic mobility shift assay (EMSA) probes and competitors. The bold and underlined bases in the EMSA probes indicate the position of rs116855380. The series of underlined bases in the transcription factor competitors indicate the site of the consensus sequence for each factor.
Table S5. Antibodies used for supershift electrophoretic mobility shift assays
