# Supplementary material for: Correlation of the Osteoarthritis Susceptibility Variants That Map to Chromosome 20q13 With an Expression Quantitative Trait Locus Operating on NCOA3 and With Functional Variation at the Polymorphism rs116855380
Source: Arthritis Rheumatol. 2015 Oct 28;67(11):2923–32. doi: 10.1002/art.39278 (PMC4832313; doi:10.1002/art.39278)
Supplement: Supplementary file 1 — Figure S1. Scatter plots of the quantitative expression of NCOA3 (left) and SULF2 (right) in osteoarthritis (OA) cartilage. Data is stratified by (A) joint site, (B) sex and (C) age at surgery. The data points represent the average of the three replicates for each sample. The expression of each gene was assessed by quantitative real‐time reverse transcription PCR and normalized to the housekeeping genes 18S, GAPDH and HPRT1. n is the number of patients studied. The horizontal lines in the columnar scatter plots represent the mean and the standard error of the mean. The trendlines in the XY scatter plots represent linear regression of the data. p‐values for (A) and (B) were calculated using a two‐tailed Mann‐Whitney exact test. p‐values for (C) were calculated using linear regression. Figure S2. Schematic representation of the 20q13 locus. Gene track is a screenshot from http://genome.ucsc.edu, using release hg19. The genes are represented by horizontal lines, with the arrows indicating the direction of gene transcription. The exons are represented by vertical bars, whose width is proportional to the length of the exon. The numbers above the gene tracks indicate the position within chromosome 20. The vertical lines beneath the gene tracks represent the SNPs analyzed in this study. The SNP that identified the signal in the GWAS, rs6094710, is labeled in white text on a black background, and the two transcript SNPs used for AEI, rs6094752 (NCOA3) and rs3810526 (SULF2), are labeled with bold text. Also labeled are the other SNPs in perfect LD with rs6094710. Figure S3. Electrophoretic mobility shift assay (EMSA) analysis in SW872 (A and B) and SW1353 cells (C and D). (A and C) Increasing concentrations of unlabeled G and A allele competitor were added to the EMSA reaction containing the G and A allele probes and nuclear extract, with the arrow indicating the specific complex binding to the probes. (B and D) The addition of increasing concentrations of HLF and TCF4 unlab [file ART-67-2923-s001.pdf]

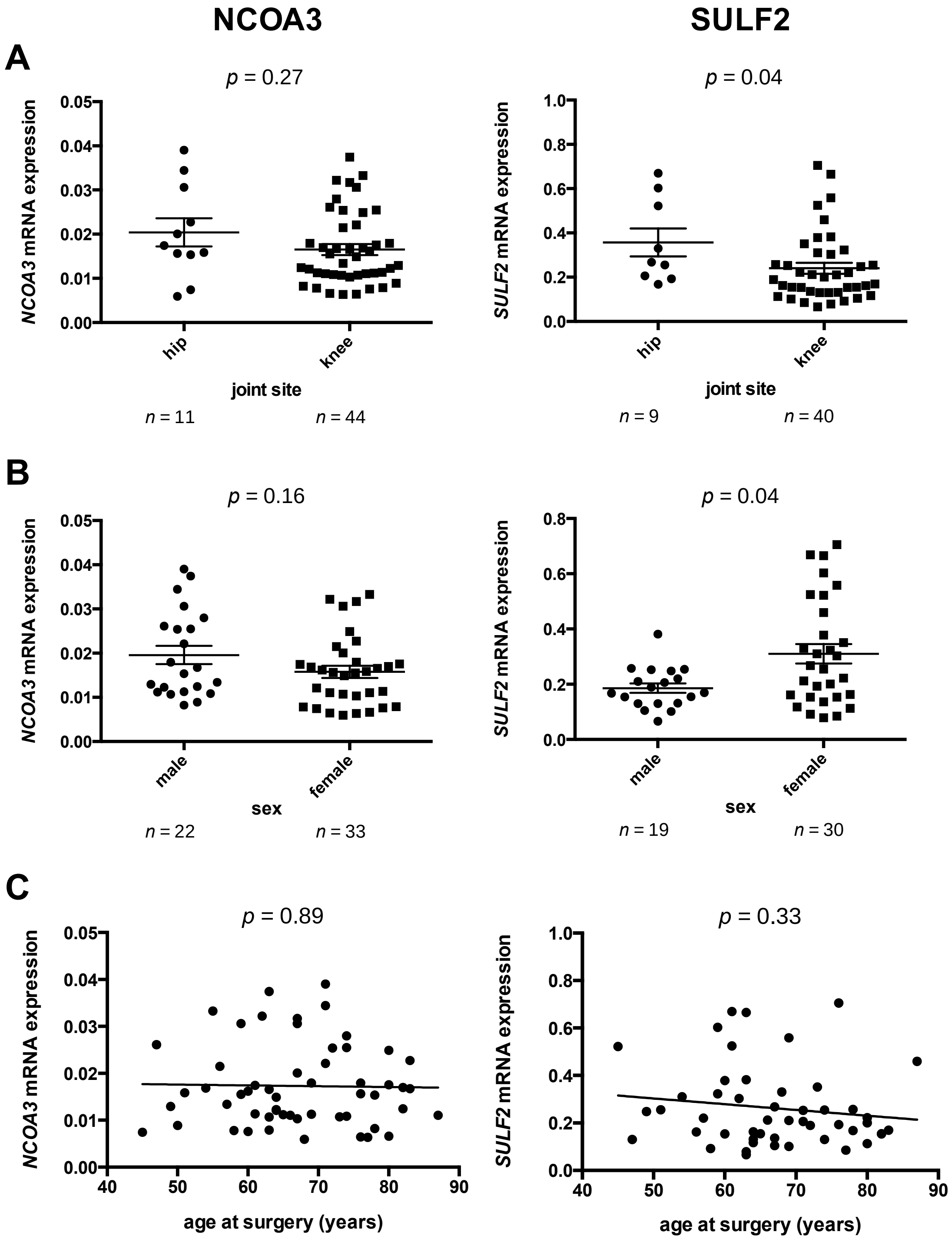

Figure S1. Scatter plots of the quantitative expression of *NCOA3* (left) and *SULF2* (right) in osteoarthritis (OA) cartilage. Data is stratified by (A) joint site, (B) sex and (C) age at surgery. The data points represent the average of the three replicates for each sample. The expression of each gene was assessed by quantitative real-time reverse transcription PCR and normalized to the housekeeping genes *18S*, *GAPDH* and *HPRT1*. *n* is the number of patients studied. The horizontal lines in the columnar scatter plots represent the mean and the standard error of the mean. The trendlines in the XY scatter plots represent linear regression of the data. *p*-values for (A) and (B) were calculated using a two-tailed Mann-Whitney exact test. *p*-values for (C) were calculated using linear regression.

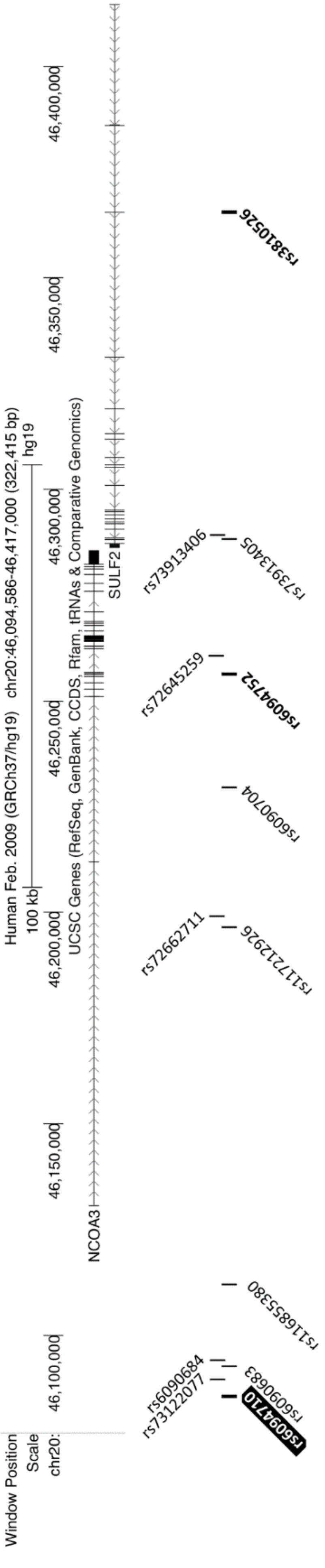

Figure S2. Schematic representation of the 20q13 locus. Gene track is a screenshot from http://genome.ucsc.edu, using release hg19. The genes are represented by horizontal lines, with the arrows indicating the direction of gene transcription. The exons are represented by vertical bars, whose width is proportional to the length of the exon. The numbers above the gene tracks indicate the position within chromosome 20. The vertical lines beneath the gene tracks represent the SNPs analyzed in this study. The SNP that identified the signal in the GWAS, rs6094710, is labeled in white text on a black background, and the two transcript SNPs used for AEI, rs6094752 (*NCOA3*) and rs3810526 (*SULF2*), are labeled with **bold** text. Also labeled are the other SNPs in perfect LD with rs6094710.

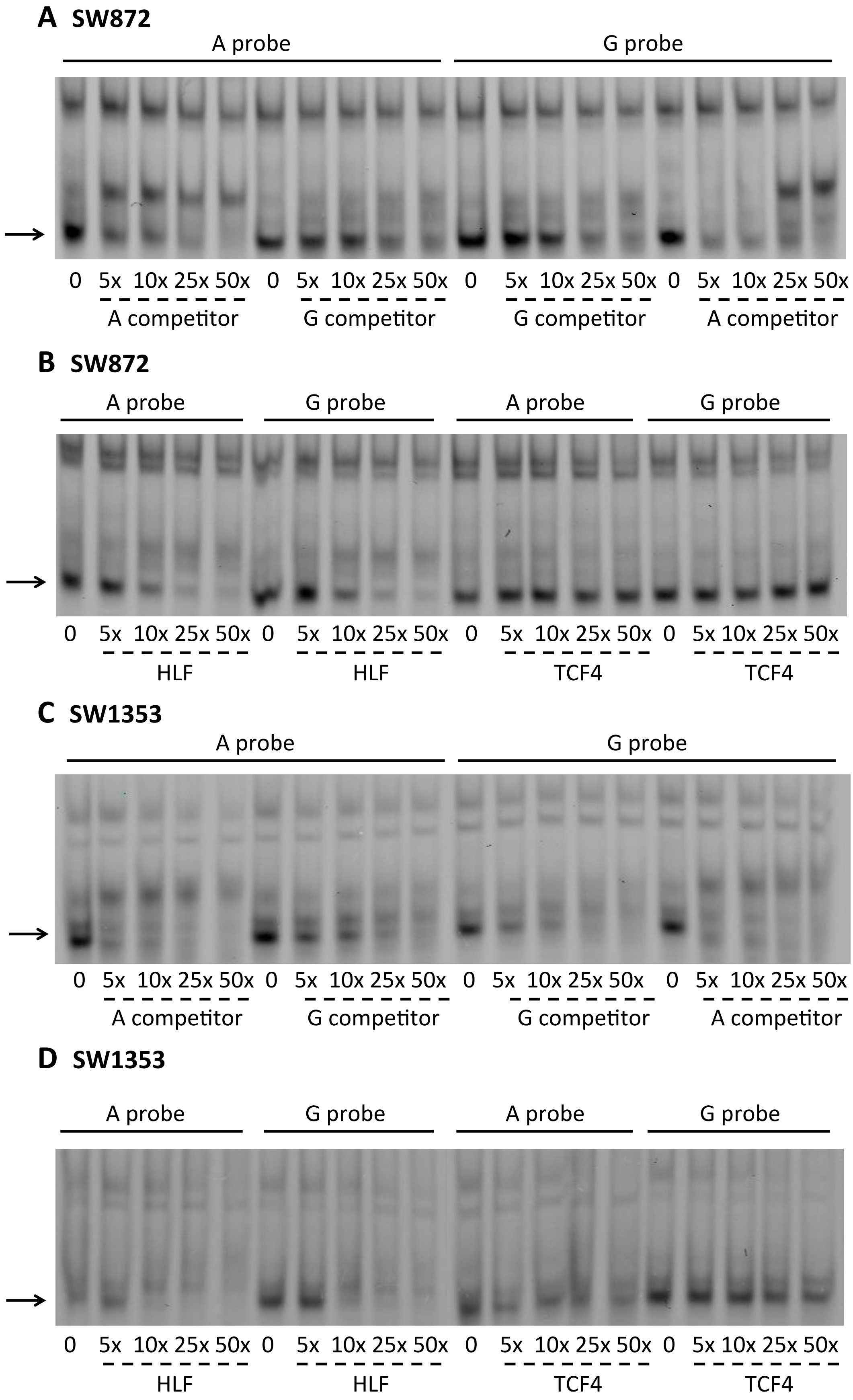
A SW872
A probe
G probe
0 5x 10x 25x 50x 0 5x 10x 25x 50x 0 5x 10x 25x 50x 0 5x 10x 25x 50x
A competitor
G competitor
G competitor
A competitor
B SW872
A probe
G probe
A probe
G probe
0 5x 10x 25x 50x 0 5x 10x 25x 50x 0 5x 10x 25x 50x 0 5x 10x 25x 50x
HLF
HLF
TCF4
TCF4
C SW1353
A probe
G probe
0 5x 10x 25x 50x 0 5x 10x 25x 50x 0 5x 10x 25x 50x 0 5x 10x 25x 50x
A competitor
G competitor
G competitor
A competitor
D SW1353
A probe
G probe
A probe
G probe
0 5x 10x 25x 50x 0 5x 10x 25x 50x 0 5x 10x 25x 50x 0 5x 10x 25x 50x
HLF
HLF
TCF4
TCF4

Figure S3. Electrophoretic mobility shift assay (EMSA) analysis in SW872 (A and B) and SW1353 cells (C and D). (A and C) Increasing concentrations of unlabeled G and A allele competitor were added to the EMSA reaction containing the G and A allele probes and nuclear extract, with the arrow indicating the specific complex binding to the probes. (B and D) The addition of increasing concentrations of HLF and TCF4 unlabeled consensus competitors to the EMSA reaction containing the G or A allele probe. The arrow indicates the complex that is competed.

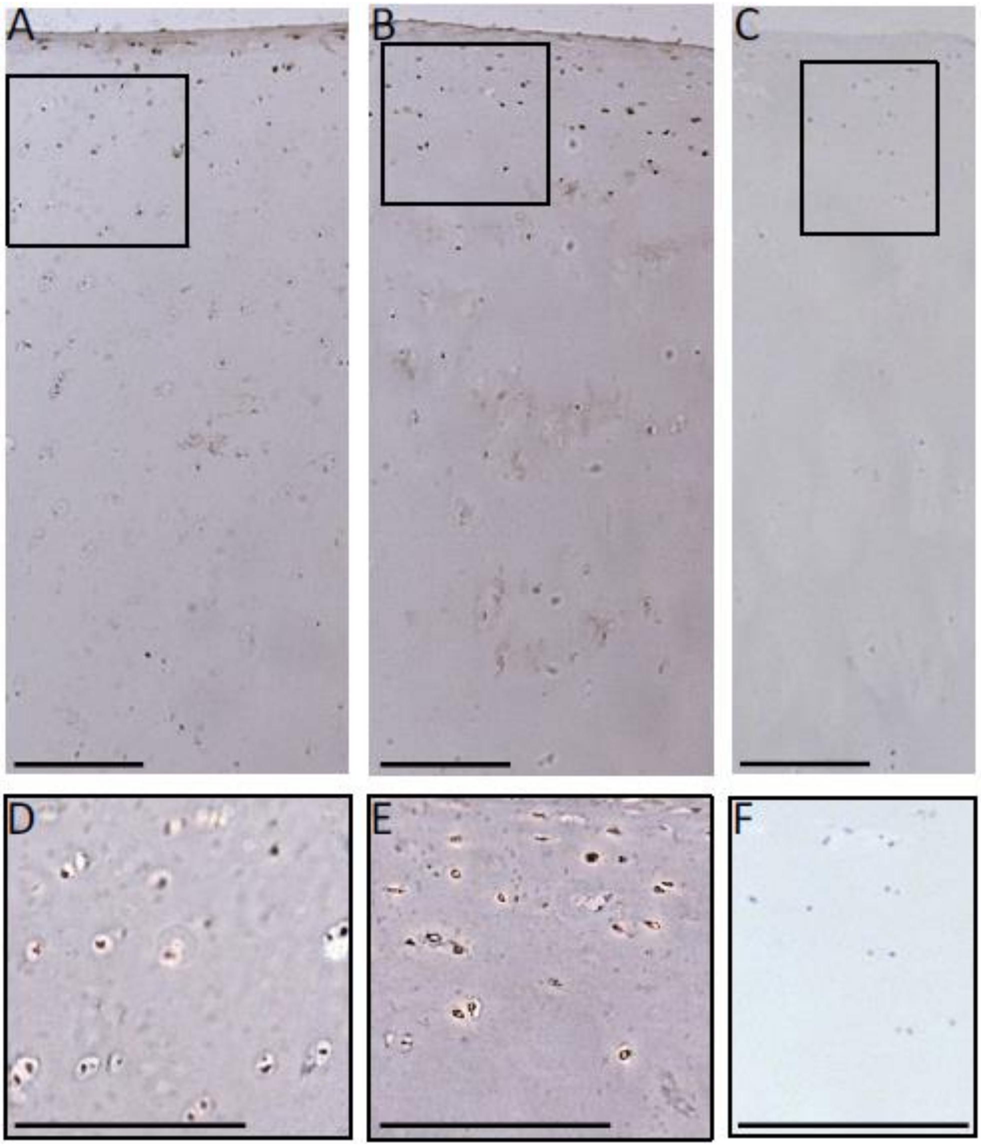

Figure S4. Expression of NCOA3 in cartilage tissue. (A,B,D,E) Immunohistochemical staining against NCOA3 in macroscopically normal knee (A and D) and hip (B and E) cartilage obtained from osteoarthritis patients. (C and F) Negative control staining with no primary antibody in the same hip sample as shown in B and E. The articular surface is at the top of the images. Top panels (A-C) were taken at 5x magnification; bottom panels (D-F) show enlarged views taken at 10x magnification. Cells exhibit nuclear staining, which is consistent with the role of NCOA3 as a co-activator of various nuclear receptors. Enlarged regions are indicated by the black boxes in A-C. Scale bars = 200 μm.

Table S1. Table of OA patient characteristics, their genotype at rs6094710 and details of their use in quantitative real-time reverse transcription PCR (RT-PCR). F, female; M, male; K, knee; H, hip.

Patient	Sex	Age at surgery (years)	Joint replacement at surgery	rs6094710 genotype	Used for RT-PCR?	
					NCOA3	*SULF2*
1	F	67	K	GG	Yes	No
2	M	71	H	GG	Yes	No
3	F	67	H	GA	Yes	Yes
4	M	74	K	GG	Yes	No
5	F	83	H	GG	Yes	No
6	M	50	K	GG	Yes	No
7	M	76	K	GG	Yes	No
8	F	55	K	GG	Yes	No
9	F	63	K	GG	Yes	Yes
10	M	57	K	GG	Yes	Yes
11	M	69	K	GA	Yes	Yes
12	M	74	K	GG	Yes	Yes
13	M	47	K	GG	Yes	Yes
14	F	69	K	GA	Yes	Yes
15	M	63	K	GG	Yes	Yes
16	F	61	H	GG	Yes	Yes
17	F	60	K	GA	Yes	Yes
18	F	66	K	GG	Yes	Yes
19	M	82	K	GG	Yes	Yes
20	F	54	K	GG	Yes	Yes
21	F	64	K	GG	Yes	Yes
22	M	63	K	GG	Yes	Yes
23	M	67	K	GG	Yes	Yes
24	F	80	K	GG	Yes	Yes
25	F	67	K	GG	Yes	Yes
26	M	64	K	GG	Yes	Yes
27	F	80	K	GG	Yes	Yes
28	F	64	K	GG	Yes	Yes
29	F	61	K	GA	Yes	Yes
30	F	80	K	GG	Yes	Yes
31	F	59	K	GG	Yes	Yes
32	M	74	K	GG	Yes	Yes
33	M	72	K	GG	Yes	Yes
34	M	71	H	GG	Yes	Yes
35	F	82	K	GG	Yes	Yes
36	M	71	K	GG	Yes	Yes
37	F	73	K	GA	Yes	Yes

38	M	69	K	GG	Yes	Yes
39	F	58	K	GG	Yes	Yes
40	F	62	K	GG	Yes	Yes
41	F	59	H	GG	Yes	Yes
42	F	63	K	GG	Yes	Yes
43	M	78	K	GG	Yes	Yes
44	M	65	K	GG	No	No
45	F	68	H	GG	No	No
46	M	63	K	GG	No	No
47	M	49	K	GG	Yes	Yes
48	M	83	K	GG	Yes	Yes
49	F	60	K	GG	Yes	Yes
50	M	65	K	GA	Yes	Yes
51	F	70	K	GG	No	No
52	F	76	K	GA	Yes	Yes
53	F	76	H	GA	Yes	Yes
54	F	63	K	GG	No	No
55	F	68	K	GG	No	No
56	M	78	H	GA	Yes	Yes
57	F	70	H	GG	No	No
58	F	45	H	GA	Yes	Yes
59	F	68	H	GA	Yes	Yes
60	F	87	K	GA	Yes	Yes
61	F	56	K	GA	Yes	Yes
62	F	77	K	GA	Yes	Yes
63	M	73	H	GG	No	No
64	F	51	H	GG	Yes	Yes
65	F	61	H	GG	No	No

Table S2. Primer and probe sequences used for the quantitative real-time reverse transcription PCR (RT-PCR) of a panel of genes. The *NCOA3* primers are not listed and were purchased from Applied Biosystems as an off-the-shelf TaqMan Gene Expression Assay. Also listed are the primers used for the pyrosequencing analysis of SNPs rs6094710, rs6094752 and rs3810526. FP, forward primer; RP, reverse primer; SP, sequencing primer; Pr, probe

RT-PCR assay	Primer sequence (5'-3')
Gene	
SULF2	FP: TGTCATTGTCTCTCTTGTGTAGC
	RP: AATCCATCCTCAAGCTGCTG
	Pr: CCCGCCAGACCCTCATCTTCTTT
COL1A1	FP: CCCCTGGAAAGAATGGAGATG
	RP: TCCAAACCACTGAAACCTCTG
	Pr: TTCCGGGCAATCCTCGAGCA
COL2A1	FP: ACCTTCATGGCGTCCAAG
	RP: AACCAGATTGAGAGCATCCG
	Pr: AGACCTGAAACTCTGCCACCCTG
ACAN	FP: TGTGGGACTGAAGTTCTTGG
	RP: AGCGAGTTGTCATGGTCTG
	Pr: CTGGGTTTTCGTGACTCTGAGGGT
SOX9	FP: CTGGTACTTGTAATCCGGGTG
	RP: ACTTGCACAACGCCGAG
	Pr: TCTGGAGACTTCTGAACGAGAGCGA
TIMP1	FP: TTCTGCAATTCCGACCTCG
	RP: TCATAACGCTGGTATAAGGTGG
	Pr: TTGACTTCTGGTGTCCCCACGAAC
RUNX2	FP: TGAAGACGGTTATGGTCAAGG
	RP: AGCAAGGTTCAACGATCTGAG
	Pr: CGGAGTGGACGAGGCAAGAGTTTC
ADAMTS5	FP: CAAGTGCGGAGTATGTGGAG
	RP: GTCTTTGGCTTTGAACTGTCG
	Pr: TTTATGTGGGTTGCCCCTTCAGGA
MMP1	FP: AAGATGAAAGGTGGACCAACAATT
	RP: CCAAGAGAATGGCCGAGTTC
	Pr: CAGAGAGTACAACTTACATCGTGTTGCGGCTC
MMP13	FP: AAATTATGGAGGAGATGCCCATT
	RP: TCCTTGGAGTGGTCAAGACCTAA
	Pr: CTACAACTTGTTTCTTGTTGCTGCGCATGA
HPRT1	FP: TGCTGAGGATTTGGAAAGGG
	RP: ACAGAGGGCTACAATGTGATG
	Pr: AGGACTGAACGTCTTGCTCGAGATG
GAPDH	FP: ACATCGCTCAGACACCATG
	RP: TGTAGTTGAGGTCAATGAAGGG

	Pr: AAGGTCGGAGTCAACGGATTTGGTC
H18S	FP: CGAATGGCTCATTAAATCAGTTATGG
	RP: TATTAGCTCTAGAATTACCACAGTTATCC
	Pr: TCCTTTGGTCGCTCGCTCCTCTCCC

**Pyrosequencing assay**	**Primer sequence (5'-3')**
SNP	
rs6094710	FP: biotin-GATTTTTCACTGGGGATGGG
	RP: CAAGTGAGCACGTACACAACTCC
	SP: CACAACTCCAATAAACACAT
rs6094752	FP: biotin-TCCTGGACAAATGAGACCCA
	RP: TCCTCCATCATAGCTCGTGG
	SP: GCATTGTTTCATATCTCTG
rs3810526	FP: biotin-GGCGAGGAGCAGTTCTCATT
	RP: CACGCTCCTCCATCCTCA
	SP: GCTCCTCCATCCTCAC

Table S3. The twelve DNA fragments examined by luciferase analysis. Chromosome locations based on UCSC genome browser, release hg19. The sites in the cloning primers for the restriction enzymes MluI (ACGCGT) and XhoI (CTCGAG) are highlighted in bold.

SNP	Alleles (major/ minor)	Chr 20 location (bp)	Primers used to clone SNP into pGL3-Promoter vector (5'-3')	Amplicon size (bp)
rs6094710	G/A	46095649	GGGG**ACGCGT**TCAAGTGATCCTCCCACCTC<br>GGGG**CTCGAG**CACACACCAGGAATGTCAGG	564
rs73122077	C/T	46099108	GGGG**ACGCGT**CGGATCACAAGGTCAGGAGA<br>GGGG**CTCGAG**ACCACACCTGGCCCATACAT	264
rs6090683	G/C	46102010	GGGG**ACGCGT**CCATAGAGCCCACAGCCTAA<br>GGGG**CTCGAG**TCAGCTCACTGCAACCTCTG	524
rs6090684	G/A	46103397	GGGG**ACGCGT**TGAGAGGGAGTCTTGCACTG<br>GGGG**CTCGAG**TGTTACAAGCTGGATGTGGTG	637
rs116855380	A/G	46120294	GGGG**ACGCGT**GTCAGGCTGGTCTCGAACTC<br>GGGG**CTCGAG**AAAAAGTTGTGGATACATTGTAGGTG	279
rs117212926	G/A	46199500	GGGG**ACGCGT**AAAAGCAGGGAGCAACTCCT<br>GGGG**CTCGAG**CATTAACGGTGCACGTGGT	482
rs72662711	T/C	46201921	GGGG**ACGCGT**TGGCCCATTTCTAGCTTTTG<br>GGGG**CTCGAG**GATCTCAGCTCTCGGCTCAC	523
rs6090704	G/A	46230204	GGGG**ACGCGT**GTGGGAGCAAGAACTCAAGG<br>GGGG**CTCGAG**CCCACCAGCTGCATGTAAT	558
rs6094752	C/T	46256424	GGGG**ACGCGT**TTGCCAGCCTGAAATGTTAGT<br>GGGG**CTCGAG**GCGTGCCACACAGATCATAC	528
rs72645259	T/C	46260639	GGGG**ACGCGT**TGTCCCTGCTGGCTTACTTT<br>GGGG**CTCGAG**CATTTCCGGATTTCTTGAGC	512
rs73913405	C/T	46288255	GGGG**ACGCGT**TGAGAACAAACAAAGGGTGGT<br>GGGG**CTCGAG**GTTGGGGTTGTTTTGCCTAA	505
rs73913406	T/G	46289063	GGGG**ACGCGT**CAGGCATGAACATCTGTGCT<br>GGGG**CTCGAG**TTTAAGGTCCCTTGGGGAAG	503

Table S4. Electrophoretic mobility shift assay (EMSA) probes and competitors. The bold and underlined bases in the EMSA probes indicate the position of rs116855380. The series of underlined bases in the transcription factor competitors indicate the site of the consensus sequence for each factor.

Probe	Primer sequence (5'-3')
DY-682 EMSA probes	
rs116855380 A allele	TATTTGTAACACAAA**A**AAAGGCCTATTATAC
	GTATAATAGGCCTTT**T**TTTGTGTTACAAATA
rs116855380 G allele	TATTTGTAACACAAA**G**AAAGGCCTATTATAC
	GTATAATAGGCCTTT**C**TTTGTGTTACAAATA
Consensus competitors	
TCF-4	CCACCGTAGTTCAAAGGATCGCACGCCCCAT
	ATGGGGCGTGCGATCCTTTGAACTACGGTGG
LEF-1	CCACCGTAGATCAAAGGATCGCACGCCCCAT
	ATGGGGCGTGCGATCCTTTGATCTACGGTGG
HLF	CCACGGTTACGTAATAGATCGCACGCCCCAT
	ATGGGGCGTGCGATCTATTACGTAACCGTGG
SRF	CCACCGTATGACCAAATAAGGCAAGCCCCAT
	ATGGGGCTTGCCTTATTTGGTCATACGGTGG
MEF-2C	CCACCGTAGGCAGTACGATCGCACGCCCCAT
	ATGGGGCGTGCGATCGTACTGCCTACGGTGG

Table S5. Antibodies used for supershift electrophoretic mobility shift assays

Antibody name	Company	Catalogue number
IgG from rabbit serum	Sigma	I5006
TCF4 (H-125)	Santa Cruz Biotechnology	sc-13027 X
HLF (H-71)	Santa Cruz Biotechnology	sc-367607
C/EBP β (C-19)	Santa Cruz Biotechnology	sc-150 X
DABP (H-40)	Santa Cruz Biotechnology	sc-98411 X
